# The role of vasculature in bone development, regeneration and proper systemic functioning

**DOI:** 10.1007/s10456-017-9541-1

**Published:** 2017-02-13

**Authors:** Joanna Filipowska, Krzysztof A. Tomaszewski, Łukasz Niedźwiedzki, Jerzy A. Walocha, Tadeusz Niedźwiedzki

**Affiliations:** 1 Chair of Anatomy, Faculty of Medicine, Jagiellonian University Medical College, 12 Kopernika St., 31-034 Kraków, Poland; 2 Department of Orthopedics and Physiotherapy, Faculty of Health Sciences, Jagiellonian University Medical College, 19e Kopernika St., 31-501 Kraków, Poland

**Keywords:** Bone vasculature, Angiogenesis and osteogenesis, Bone development and regeneration, Type H and L endothelial cells, Bone vasculature impairment, Skeletal and systemic diseases

## Abstract

Bone is a richly vascularized connective tissue. As the main source of oxygen, nutrients, hormones, neurotransmitters and growth factors delivered to the bone cells, vasculature is indispensable for appropriate bone development, regeneration and remodeling. Bone vasculature also orchestrates the process of hematopoiesis. Blood supply to the skeletal system is provided by the networks of arteries and arterioles, having distinct molecular characteristics and localizations within the bone structures. Blood vessels of the bone develop through the process of angiogenesis, taking place through different, bone-specific mechanisms. Impaired functioning of the bone blood vessels may be associated with the occurrence of some skeletal and systemic diseases, i.e., osteonecrosis, osteoporosis, atherosclerosis or diabetes mellitus. When a disease or trauma-related large bone defects appear, bone grafting or bone tissue engineering-based strategies are required. However, a successful bone regeneration in both approaches largely depend**s** on a proper blood supply. In this paper, we review the most recent data on the functions, molecular characteristics and significance of the bone blood vessels, with a particular emphasis on the role of angiogenesis and blood vessel functioning in bone development and regeneration, as well as the consequences of its impairment in the course of different skeletal and systemic diseases.

## Vasculature of the bone: the role in skeleton development, remodeling and regeneration

Bone is a highly vascularized connective tissue. Skeletal vasculature plays a significant role in the process of bone development (endochondral and intramembranous ossification), regeneration and remodeling [1–3]. The skeletal system receives between 10 and 15% of total cardiac output [4]. Not only do blood vessels supply the skeletal system with oxygen or nutrients and remove metabolites from the bone, they also provide the skeleton with specific hormones, growth factors and neurotransmitters secreted by other tissues (e.g., brain-derived serotonin, [5]), maintaining the bone cells survival and stimulating their activity [6].

The significance of appropriate interactions between the blood vessels and bone cells is well illustrated by different abnormalities, leading to development of some skeletal diseases, e.g., craniofacial dysmorphology [7, 8] or idiopathic osteonecrosis [9]. Both diseases result from inappropriate angiogenesis either during development of the skeleton or an abnormal vascular system functioning within the mature bones.

**Fig. 1:**
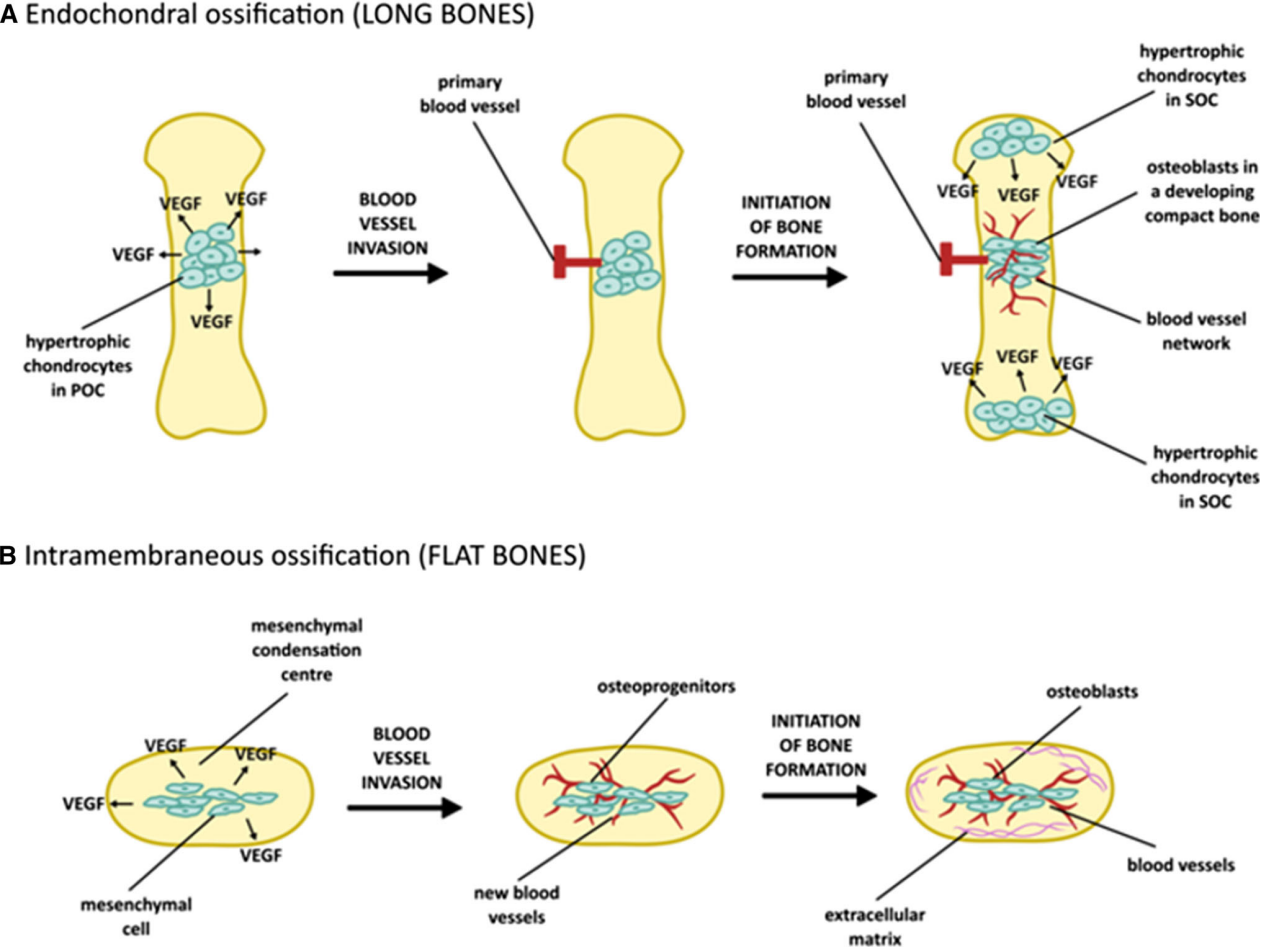
The stages of blood vessel invasion during endochondral **a** and intramembraneous **b** ossification. *VEGF* vascular endothelial growth factor, *POC* Primary Ossification Center, *SOC* Secondary Ossification Center. Based on [12, 20]

Bones containing osteoblasts which terminally differentiate into osteocytes develop from a mesenchymal stem cell precursor which can undergo osteogenesis through two distinct mechanisms [10]. Endochondral ossification (with cartilage as an intermediate stage) is typical for the long bones, e.g., femur or tibia, whereas intramembranous ossification (related to a direct differentiation of mesenchymal stem cells into osteoblasts) is the main mechanism leading to a development of flat (e.g., craniofacial) bones [11]. In both modes of osteogenesis, angiogenesis, associated with the production of vascular endothelial growth factor (VEGF) by either hypertrophic chondrocytes or differentiating mesenchymal cells, constitutes a critical stage [12] (Fig. 1).

Secretion of VEGF is essential for coupling of osteogenesis and angiogenesis. VEGF (particularly VEGF-A) acts as a chemotactic molecule, attracting endothelial cells toward a bone tissue and directly controlling differentiation and functions of osteoblasts and osteoclasts, participating in the bone remodeling [13]. As shown by Maes et al. [14], two main isoforms of the mouse VEGF-A, namely VEGF_164_ and VEGF_188_ (equivalents of the human VEGF_165_ and VEGF_189_ [15]) determine a proper bone formation through the endochondral ossification. A complete loss of both of these isoforms leads to an impaired bone vascularization, growth plate morphogenesis and disturbed endochondral ossification.

This is specifically caused by an impaired differentiation and activity of the hypertrophic chondrocytes, but also osteoblasts, endothelial cells and osteoclasts, critical for bone formation and functioning. Maes et al. [16] also found that among the two mouse-specific isoforms of the VEGF-A: VEGF_164_ and VEGF_188,_ only VEGF_164_ expressed alone can compensate the loss of the VEGF_188_ isoform, without any detrimental effects on proper bone development. On the other hand, VEGF_188_ isoform expressed alone is insufficient to properly direct the proliferation and survival of chondrocytes under hypoxic conditions, indispensable for an establishment of the epiphyseal vascularization during endochondral bone formation.

In another paper, Maes et al. [17] showed that in mice VEGF_164_ activates the PI3K/AKT pathway in osteoblasts and induces a stabilization and signal transduction by the main component of the Wnt signaling pathway, β-catenin. Overexpression of VEGF_164_ isoform leads to osteosclerosis, highly increased bone formation, accompanied by an intensified osteoblast differentiation resulting in bone overgrowth and altered bone morphology. This indicates a need for a tight angiogenesis control during bone development. Similar data have recently been published by Ben Shoham et al. [18]. Interestingly, recent data also point to the role of other molecules secreted by bone blood vessel endothelial cells, e.g., Noggin (for further details, see Chapter 3).

During bone development by the endochondral ossification, cartilage vascularization begins with the formation of hairpin loops, projecting from the perichondrial vascular network to the adjacent cartilage. Next, a capillary glomerulus forms at the leading edge and the entire vascular unit grows in length with a mushroom-like shape. Following elongation, the vascular unit is accompanied by a backward expansion of the capillary network, tightly surrounding a pair of main vessels (arteriole and venule) [19].

Blood vessel formation during intramembraneous ossification (Fig. 1) was first described for a frontal bone of a chick embryo by Thompson et al. [20]. These authors demonstrated that during a developmental period, the capillaries of a small diameter move into the thin avascular layer of loose mesenchyme, which surrounds the mesenchymal condensation center, where mesenchymal cells secrete VEGF, attracting endothelial cells (Fig. 1). This process takes place shortly before the initial ossification of the frontal bone. Then, the small vessels invade the condensation center right at or nearby the site of initial ossification, at the supra-orbital ridge. Shortly afterward, bone, which first undergoes mineralization, associates with an extensive internal and external network of blood vessels. Further cascade of vascular invasion and ossification continues as a front of bone expansion moves externally in all directions. Osteoblasts further participating in bone mineralization, lay down at the vicinity of capillaries possessing a large diameter.

Blood vessels also play a central role in the process of bone remodeling and regeneration. During cortical bone remodeling, the cutting cone, built of osteoclasts, moves forward and resorbs either a dead bone or a damaged/old bone matrix. The cutting cone is followed by a blood vessel, which delivers nutrients and growth factors for osteoblasts to lay down new bone behind the cone [5]. However, a question remains whether these are osteoblasts or any other types of bone-forming cells, directly following the blood vessels in developing or regenerating/remodeled bones. The report by Maes et al. [21] indicates the specific involvement of the osterix-expressing osteoblast precursors, accompanying the blood vessels during endochondral bone development and regeneration. The authors revealed that some of these precursors exhibit a perivascular localization and directly associate with the invading blood vessels, which confirms a strong relationship between angiogenesis and osteogenesis, but also indicates that these are not mature osteoblasts, directly following the bone-invading blood vessels (for further details, see Chapter 3).

There are two basic mechanisms of blood vessel formation: vasculogenesis (formation of a blood vessel from a progenitor cell, angioblast or hemangioblast) [22] and angiogenesis (new vasculature development from the preexisting blood vessels) [23]. Vasculogenesis is critical for the proper development of a whole embryo and as such indirectly leads to the establishment of the primary bone vasculature, through angiogenesis [24], which may for instance rely on the bone-endothelium specific Notch signaling and Nogging secretion (for further details see Chapter 3). Angiogenesis also occurs during bone regeneration [25].

The main source of different growth and differentiation factors, primarily VEGF, which is indispensable for angiogenesis within a developing or mature bone tissue is hypertrophic chondrocytes, mesenchymal cells or the blood vessel-localized endothelial cells [26]. However, a recent study by Wiszniak et al. [27] shows that during a development of the flat bones, cranial neural crest cells (NCCs) and the NCC-derived cells may also constitute an essential source of VEGF which supports the blood vessel growth in the developing jaw. Mice lacking the production of VEGF in NCC-derived tissue exhibit a mandibular hypoplasia. Therefore, according to Wiszniak et al. [27], undisrupted secretion of VEGF is critical for normal chondrocyte proliferation and Meckel’s cartilage growth as well as for further jaw extension. Bone cells themselves also are a vital source of VEGF (for further details, see the Chapter 3).

## Vasculature of the bone: its organization and involvement in the process of hematopoiesis

**Fig. 2:**
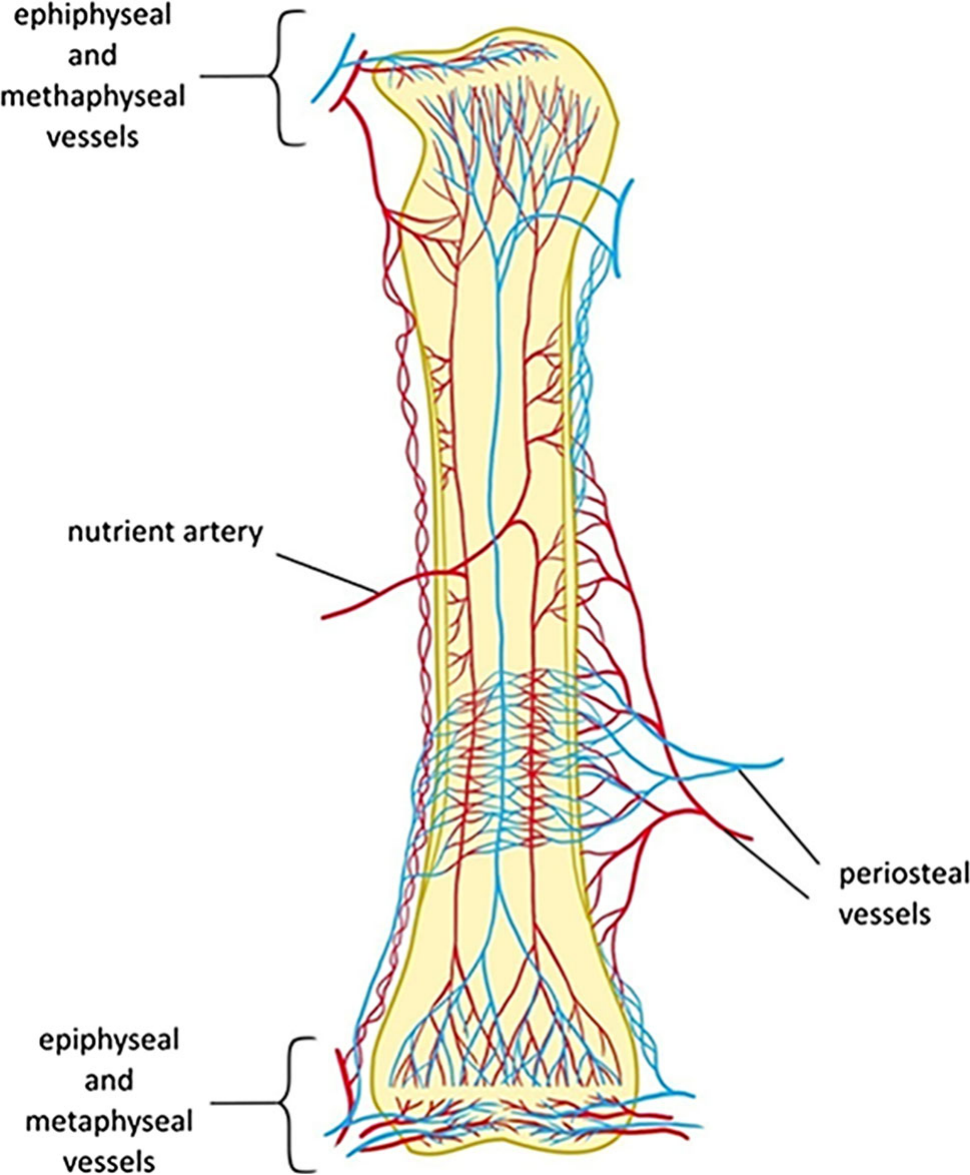
Vascular system supplying the long bones. Based on [30]. Arteries/arterioles are marked in red and veins/venules in *blue*

The presence of a blood vessel network within bone was first discovered in the seventeenth century in the experiments of van Leewenhoek, to be further confirmed and extensively researched in the twentieth century by the groups of Trueta et al. [28, 29], Brookes [30], Thompson et al. [20], Skawina et al. [31–33] or Georgia et al. [34]. The experiments of Brookes showed that blood circulates within the bone cortex in a centrifugal and not in a centripetal direction [30]. This means that blood flows inside the cortical bone through the nutrient arteries in the marrow cavity and is returned by the periosteal veins [35] (Fig. 2). However, a centripetal flow can replace a centrifugal flow as a result of trauma or metabolic disease [36].

Studies of the bone-localized vasculature and microvasculature possess significant clinical relevance, particularly in the context of bone regeneration approaches in facial surgery [38]. Previously, the bone vascular system was analyzed mostly via India ink injections [37]. Currently, the macroscopic organization of the vascular network within bones is additionally studied by means of scanning electron microscopy (SEM) vessel corrosion-casts or computed tomography methods [19, 38–40].

At the macroscopic level, the blood vessel network supplying the long bones is composed of three main systems: (1) the central nutrient artery, (2) metaphyseal-epiphyseal arteries, and (3) periosteal artery [41]. It is worth mentioning that in the long bones, the central nutrient artery supplies the entire medullar cavity and the 2/3 of the outer cortical bone, whereas the periosteal arteries penetrate the other 1/3 of the outer cortical bone (Fig. 2).

At the microscopic level, blood vessels of the long bones localize within the Haversian (central) and Volkmann’s (perforating) canals in compact bone and further pass above the medullary cavity, through the trabeculae of the spongy bone [42]. In both Haversian and Volkmann’s canals, blood vessels are accompanied by nerves.

Other types of bones: flat or irregular are differently supplied with vasculature. Flat bones, i.e., cranial bones, are delivered with blood through the periosteal arteries [43]. Mandible (an example of the irregular bone) is supplied by three arteries: the lingual, facial and inferior alveolar arteries. The first 2 arise directly from the external carotid, a major artery [44].

The microvasculature of, e.g., the long and flat bones possesses some distinct characteristics. For example, Pannarale et al. [45] analyzed the microvasculature in rat parietal, scapula, and ileum bone by means of vascular corrosion cast (vcc, a resin-cast preparation), using the light and SEM microscopy. The authors observed that the bone thickness strongly affects the pattern of the microcirculation in the flat bones and that it varies in the thick and thin parts of all types of bones mentioned above. At the sites thinner than 0.4 mm, only the periosteal and dural networks exist. Larger vessels, which do not form a true vascular network, connect the two sites of the bones in these regions. In thicker sites, the organization of the microvasculature is similar to that observed in the long bones. There, distinct periosteal, cortical and bone marrow networks exist. Moreover, depending on the bone type and surroundings (dura mater, muscles etc.), outer networks exhibit moderate changes in their characteristics. Several types of vessels were recognized by comparing their different diameter, course and endothelial imprints. The observations of Pannarale et al. may be explained by the variable metabolic and blood flow-originating needs of different bones and a great influence of the bone architecture itself on the formation of the blood vessel network.

Besides secreting different nutritional and signaling factors, e.g., VEGF-A, blood vessels localized in the bone marrow cavity orchestrate the process of hematopoiesis, and provide the Hematopoietic Stem Cells (HSCs) with the necessary niche. This has been extensively researched particularly within the long bones, e.g. [46].

Although it is currently commonly accepted that HSCs are localized at the vicinity of bone blood vessels (both arteries and veins), a specific location of their niche has been unclear so far [47, 48]. However, recently Acar et al. [47] have performed the analyses of the mouse HSC niche localization within the bone marrow of the long bones (tibiae and femurs). They have found that approximately 85% of cells are placed within 10–30 μm of a sinusoidal blood vessel. Most HSCs, both dividing (defined as Ki-67(+)) and non-dividing (defined as Ki-67(−)), localize in the perisinusoidal niches with Lepr(+)Cxcl12(high) cells throughout the bone marrow and are not positioned at the vicinity of the arterioles, transition zone vessels, or bone surfaces. On the other hand, Kunisaki et al. [48] demonstrated that quiescent HSCs associate specifically with the small arterioles, very commonly distributed within the endosteal bone marrow, whereas in a study by Kusumbe et al. [49] HSCs were frequently detected near the type H endothelium (for further details see Chapter 3) and arterioles in the endosteal region. Itkin et al. [50] found that distinct types of blood vessel with different permeability properties regulate mammalian bone marrow stem cell maintenance and leukocyte trafficking. Arterial blood vessels of a lower permeability (containing the Sca-1-, VE-cadherin- and nestin-positive cells) maintain haematopoietic stem cells in a low reactive oxygen species (ROS)-inactive state, whereas the sinusoids of a higher permeability (VE-cadherin-positive) promote hematopoietic stem and progenitor cells (HSPCs) activation and constitute the niche for immature and mature leukocyte within the bone marrow.

These results altogether suggest that the arteriolar niches are responsible for maintaining HSC quiescence. A continuous interchange between the two niches provides a balance in HSCs proliferation and quiescence, which, when disrupted, can lead to a development of leukemia [48]. The knowledge resulting from those studies may also be critical for the elaboration of HSCs mobilization and transplantation protocols [50]. Furthermore, the studies described above show that bone blood vessels, as the orchestrators of hematopoiesis, play an important systemic role in maintaining the body homeostasis.

## The role of blood vessel endothelium specialization during bone formation and regeneration: significance of VEGF-A secretion by osteoblasts

Even though blood vessels are constituted of many types of cells, the main secretory role in angiogenesis is assigned to endothelial cells. The results of the studies by Kusumbe et al. [51] and Ramasamy et al. [52] suggest that blood vessels of the bone (namely the arterioles) are lined with a distinct type of the specialized endothelial cells, permeable to some growth factors that specifically stimulate differentiation of the bone precursor cells.

The previously mentioned authors, Kusumbe et al. [51], identified two types of endothelial cells in the blood vessels of the bones- type H and L. These two types differ in terms of the surface marker expression and the level of expression of HIF1-α (hypoxia-inducible factor 1 alpha), a transcription factor stimulating the growth of new blood vessels through angiogenesis. Type H vascular endothelial cells express higher levels of HIF1-α and are localized in the vicinity of the chondro–osseous junction. They also exhibit an intense CD31/endomucin (CD31(hi)Emcn(hi)) expression. Type L endothelial cells are localized in the sinusoidal vessels in the diaphysis and are characterized by a low expression of CD31 and endomucin (CD31(lo)) [51, 53]. Contrary to type H, the number of type L endothelial cells does not change over time, which was demonstrated by Kusumbe et al. [51] in the experiments on young and old mice. These studies indicated that the number of type H endothelial cells is age-dependent and critical to appropriate bone functioning [51].

**Fig. 3:**
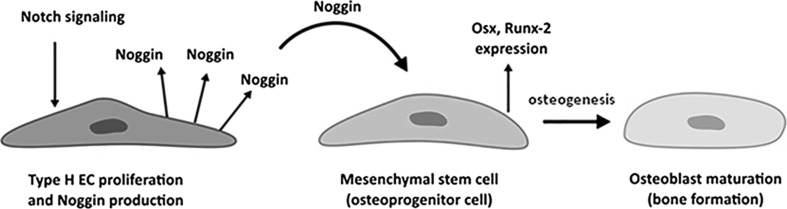
Molecular crosstalk between type H endothelial cells (Type H EC) and mesenchymal stem cells, leading to osteoblast maturation and final bone formation. Based on [51]

Kusumbe et al. [51] also showed that the precursor cells involved in bone regeneration interact mostly with the type H endothelial cells. According to those authors, an age-related loss of bone precursor cells documented in a murine model is associated with a gradual decrease in the number of type H endothelial cells. This might explain a diminished regeneration potential of the bone, observed in the elderly. In turn, the findings published by Ramasamy et al. [52] suggest that maturation of type H endothelial cells involves the Notch signaling pathway, while a blockade of the latter cascade results in an impairment of bone formation in a murine model, its activation promotes osteogenesis. The authors have demonstrated that Notch activation in ECs is required for the endothelial cell proliferation and stimulation of Noggin production (Fig. 3). Endothelial Notch pathway and Noggin regulate differentiation of perivascular osteoprogenitor cells and through this osteogenesis, as well as promote the chondrocyte maturation and hypertrophy. This affects angiogenesis through the expression of VEGF-A by the hypertrophic chondrocytes. The results of these studies show that blood vessels of the bone, although mostly similar in function to the vascular network of other tissues, possess unique characteristics relying on the Notch/Noggin signaling which enable their specific interaction with the bone cells [52]. Specialization of the bone blood vessel endothelium described above was also observed in some bone tissue engineering-based experiments (for further information, see Chapter 5). For instance, recent studies by Eman et al. [54] have shown that ectopic bone formation is coupled with a specific type of peripheral blood-derived endothelial progenitor cells (EPCs), the so-called early-outgrowth EPC, which, based on 3D matrigel plugs, cooperate with mesenchymal stem cells in bone tissue-engineered construct development.

Interestingly, a recent report by Ben Shoham et al. [18] indicates a new role of bone vasculature, showing that it can serve as a guiding template for a deposition of the mineralized bone matrix. Using a mouse model, Ben Shoham et al. demonstrated that during the embryonic development, endothelial cells localized within the bone blood vessels lack a basement membrane. They are instead covered by the osteoblast-secreted collagen type I and can undergo a gradual mineralization, thus serving as a mineralization template.

As mentioned previously, endothelial cells are the most important secretory cells, producing VEGF within the blood vessels [55]. However, many reports also point to a significant role of the bone cells themselves in the regulation of bone resorption and formation through the secretion of factors typical for blood vessel endothelium. For instance, the studies by Hu et al. [13] have demonstrated that VEGF-A, produced not by endothelial cells but particularly by osteoblasts, is indispensable for the onset of bone regeneration in vivo. By using the mouse knockout model of the osteoblast-specific VEGF-A, the authors confirmed that this factor regulates bone defect regeneration through endochondral and intramembranous ossification and stimulates the resorptive activity of the osteoclasts. They also observed the existence of optimal concentrations of exogenous VEGF which enable the coupling of angiogenesis and osteogenesis in intramembranous ossification-driven bone regeneration.

VEGF-A can be expressed under hypoxic conditions, which stimulate accumulation of HIF-1α inside the osteoblasts [56]. A recent paper by Cui et al. [57] indicates a molecular mechanism of hypoxia and HIF-1α involvement in the endochondral bone development. The authors revealed that OASIS (also called CREB3L1), an endoplasmic reticulum (ER)-resident transcription factor, is upregulated in a time-dependent manner during hypoxia and binds to HIF-1α through its basic leucine zipper (bZIP) domain.

This promotes a transcription of hypoxia-inducible genes, including VEGF-A under hypoxic conditions.

The authors also found that OASIS-related deficiency in osteoblasts leads to the impairment of vessel formation in bone tissue and consequently impairs the process of bone development.

## Dysfunction of bone blood vessels in skeletal and systemic diseases

Blood vessels supplying bones can undergo significant changes when affected by both skeletal and systemic diseases (Table 1). Among the skeletal diseases, osteonecrosis, also referred to as avascular necrosis, seems to be directly associated with the blood vessel supply impairment [58]. Avascular necrosis of the femoral head (ANFH) is a pathologic process resulting from the interruption of blood supply to the femur, characterized by development of ischemic injury, necrotic death of the osteocytes, the collapse of articular surface and the eventual onset of osteoarthritis [59, 60]. The onset of this disease may be initiated by femoral neck fractures, hip dislocations, smoking or alcohol abuse. Feng et al. [60] suggested that femoral head necrosis may be associated with endothelial progenitor cells (EPCs) biology impairment, i.e., low number of those cells, their decreased capacity to migrate and increased senescence. EPCs, first characterized by Asahara et al. [61], represent a subpopulation of bone marrow-derived endothelial progenitors which may substantially contribute to a new blood vessel formation by promoting new bone growth through neovascularization, [61, 62]. Thus, a change in the numbers and functions of EPCs, accompanying the ANFH, may reflect its common underlying etiology [60]. It also is suggested that avascular necrosis stems from a damage of the endothelial cell membrane, in some circumstances combined with an increased susceptibility of an individual to the blood clot formation. This causes an interruption of the blood flow which finally leads to osteonecrosis [59].

**Table 1:** The effects of the skeletal and systemic diseases on bone vasculature functioning

Skeletal or systemic disease	The effects of a disease on bone vasculature and bone functioning in humans or laboratory animals
Avascular necrosis of the femoral head (ANFH)	Decreased neovascularization caused by low number of endothelial progenitor cells (EPCs), their diminished capacity to migrate and increased senescence in humans [56] Blood flow interruption caused by damage of the endothelial cell membrane, in some circumstances combined with an increased susceptibility of an individual to blood clot formation [55] Ischemic injury, necrotic death of the osteocytes, the collapse of articular surface and the eventual onset of osteoarthritis [55, 56]
Postmenopausal osteoporosis	Decrease in blood vessel volume and expression of angiogenesis-related HIF-1α, HIF-2α, and VEGF proteins in ovariectomized mice, suggesting that estrogen deficiency-induced bone loss is accompanied by a decrease in number of bone marrow-localized blood vessels [60]
Diabetes mellitus	Microangiopathy accompanying DM causes vasoconstriction and impairs blood flow within the long bones of ZDF rats [63] Decreased blood vessel supply, especially within bone marrow, may further lead to osteopenia, usually observed in chronic T2DM-affected long bones [63] Advanced glycation end products (AGEs), produced in diabetes mellitus, may also disrupt bone vasculature [68]
Atherosclerosis	Oxidized lipids produced during atherosclerotic plaque formation within bone blood vessels negatively affect bone mass by increasing anti-osteoblastogenic inflammatory cytokines and decreasing pro-osteoblastogenic Wnt ligands in ApoE-knockout, high-fat-diet-fed mice [70]

Also, osteoporosis, especially related to the postmenopausal period, is associated with some significant changes within the bone blood vessels. It is commonly accepted that postmenopausal osteoporosis is characterized by a reduced number of sinusoidal and arterial capillaries in the bone marrow, leading to a decreased bone perfusion [64]. Zhao et al. hypothesized that the activation of the HIF signaling pathway (as mentioned previously, the hypoxia- and HIF-related pathway is involved in angiogenesis) in mouse osteoblasts, through osteoblast-specific disruption of the HIF-degrading protein, i.e., Von Hippel–Lindau (VHL) (ΔVhl), may protect mice from the ovariectomy-induced bone loss. Compared to control littermates, ΔVhl mice, with the genetically enhanced HIF signaling in osteoblasts, had significantly increased trabecular and cortical bone volumes. The authors also observed an enhanced blood vessel formation compared to the controls. In the latter, ovariectomy significantly decreased bone mineral density, significantly affected the bone microarchitecture, i.e., caused a decrease in the bone volume/total volume ratio or tibia thickness, measured by means of the micro-CT method, and caused a decrease in mechanical strength compared to the control group (sham operated mice). Significant decrease in the blood vessel volume and expression of angiogenesis-related HIF-1α, HIF-2α, and VEGF proteins at the distal femur in ovariectomized control mice were also noted. No such effects were observed in ΔVhl mice. These results suggest that activation of the HIF signaling pathway in osteoblasts may protect from estrogen deficiency-induced bone loss and bone marrow-localized decrease in blood vessels. Also Liu et al. [65], using a rat model of ovariectomy, demonstrated that increased bone vascularity and angiogenesis in the bone marrow might protect from the bone loss, associated with the cessation of estrogen production.

Besides the skeleton-associated diseases, systemic diseases also negatively affect bone vasculature. Bone blood supply may be compromised, e.g., in type 2 diabetes mellitus (T2DM). It is commonly known that T2DM leads to the development of microangiopathy [66]. Recently, Stabley et al. [67] performed the studies on Zucker diabetic fatty (ZDF) rats, a model of T2DM, and have shown that progression of this disease causes vasoconstriction and impairs the blood flow within the long bones. The authors suggested that a decreased blood vessel supply, especially within bone marrow, may further lead to osteopenia, usually observed in chronically T2DM-affected long bones. The results of Oikawa et al. [68] suggest similar changes within the bone marrow-localized blood vessels in type 1 diabetes mellitus (T1DM). Recently, Peng et al. [69] characterized bone microstructure, strength, and the course of bone turnover in a study performed on streptozotocin (STZ)-induced diabetic mice (T1D mice). The authors also explored the role of angiogenesis in the pathogenesis of T1D-induced osteoporosis. The study revealed a decreased angiogenesis (i.e., reduced number of blood vessels in the femur and decreased expression of platelet endothelial cell adhesion molecule (CD31), NGF-nerve growth factor, the previously mentioned HIF-1α and VEGF) in type 1 diabetic mice compared to their healthy counterparts. Peng et al. [69] concluded that the impairment of angiogenesis is the main cause of a decreased bone formation and imbalanced remodeling, observed in diabetes. A report recently published by Portal-Núñez et al. [70] indicates an important role of aging in worsening the bone-related complications associated with diabetes mellitus. In this study, Portal-Núñez and co-workers used male CD-1 mice, 2 month-old (young control group) or 16 month-old (old animals). The authors analyzed two groups of mice: non-diabetic and diabetic (streptozotocin-induced), within both age groups (young vs. old). Portal-Núñez et al. observed that DM resulted in a decrease in some bone-related parameters, e.g., the bone volume, trabecular number as well as caused a diminished mineral apposition and bone formation rates. They also observed significant bone structure changes, with the increased trabecular separation within L1-L3 vertebrae of aged mice. Tibias of the old diabetic mice also exhibited changes in the three-point bending tests, indicating their increased frailty and brittleness. DM-suffering old mice also had a decreased expression of both VEGF and its type 2 receptor, which were accompanied by an impaired femoral vasculature. The authors also observed an increased expression of the pro-adipogenic gene peroxisome proliferator-activated receptor gamma (PPAR-γ) and increased adipocyte number in the bones of elderly mice. The levels and activity of the reactive oxygen species-scavenging enzymes, e.g., glutathione, remained unchanged in the old diabetic mice, with the levels of xanthine oxidase slightly increased in the bone marrow of those same mice. This may indicate that intensive adipogenesis, replaces osteogenesis within the bone marrow of the old mice. However, Portal-Núñez and co-workers [70] also noted an elevated expression of the senescence-associated marker—caveolin-1 and p-53—in the old, diabetic mice. Based on these results, the authors concluded that the bone-related complications accompanying DM are intensified during aging, and these effects are strongly associated with an imbalanced bone turnover, decreased vasculature parameters, and increased senescence marker expression, but seem to be independent of the anti-oxidative system or the Wnt signaling pathway in the bone cells.

The studies by Bandeira et al. [71] suggested a link between the iliac artery calcification and osteoporosis in type 2 diabetic male patients. The authors examined 59 diabetic men aged 50–80 years, examined using bone densitometry DXA (dual-energy X-ray absorptiometry) method. The conclusion from this study was that there is a significant association of osteoporosis in the lumbar spine L1-L4 (*p* = 0.004) and in the femoral neck (*p* = 0.036) with the iliac artery calcification, but the authors did not explore any molecular mechanisms related to this phenomenon. However, Bandeira et al. [71] suggest that it may probably be associated with the elevated levels of osteoprotegerin as well as receptor activator of Nfκβ ligand (RANK-L), usually extensively produced in diabetes mellitus by either immune cells or endothelial cells. This seems to confirm a strong relationship between bone metabolism, inflammatory status and a condition of bone vasculature. Some studies suggest a significant role of the advanced glycation end products (AGEs), produced in diabetes mellitus, in the pathophysiology of atherosclerosis (a metabolic disease affecting blood vessels, see the paragraph below) and their direct link with diabetes-related osteoporosis [72].

It is also currently believed that there exists a link between osteoporosis and non-diabetic atherosclerosis. The results of Prasad et al. [73] have indicated this association in a study performed on 194 postmenopausal osteoporosis-affected women. The authors have shown that the coronary microvascular endothelial dysfunction (CMED) is an independent predictor of osteoporosis in women aged more than 50 years. Recently, Liu et al. [74] have found a link between bone loss and atherosclerosis-resulting inflammation in ApoE-knockout mice. The authors showed that the osteoblast number and function were dramatically reduced in trabecular and cortical bone of the ApoE-knockout, high-fat-diet-fed (HFD) mice, a mouse model of atherosclerosis [75]. At the same time, the osteoclast number did not change in trabecular bone only. Consequently, the authors observed a decreased number of osteoblast progenitors and increased number of monocyte/macrophages in the bone marrow as well as increased expression of the proinflammatory cytokines IL-1β, IL-6, and TNF. High-fat diet also impaired the Wnt signaling, by inhibiting the expression of the Wnt-targeted genes and downregulated the expression of the pro-osteoblastogenic Wnt ligands. These results suggest that the oxidized lipids produced during atherosclerotic plaque formation probably also within bone blood vessels negatively affect bone mass by increasing anti-osteoblastogenic inflammatory cytokines and decreasing the pro-osteoblastogenic Wnt ligands.

This knowledge may potentially contribute to the elaboration of new strategies targeting blood vessels localized within bone, which directly affect proper functioning of the skeletal system and also the whole body.

## Angiogenesis-targeted bone tissue engineering or gene therapy in bone regeneration

**Fig. 4:**
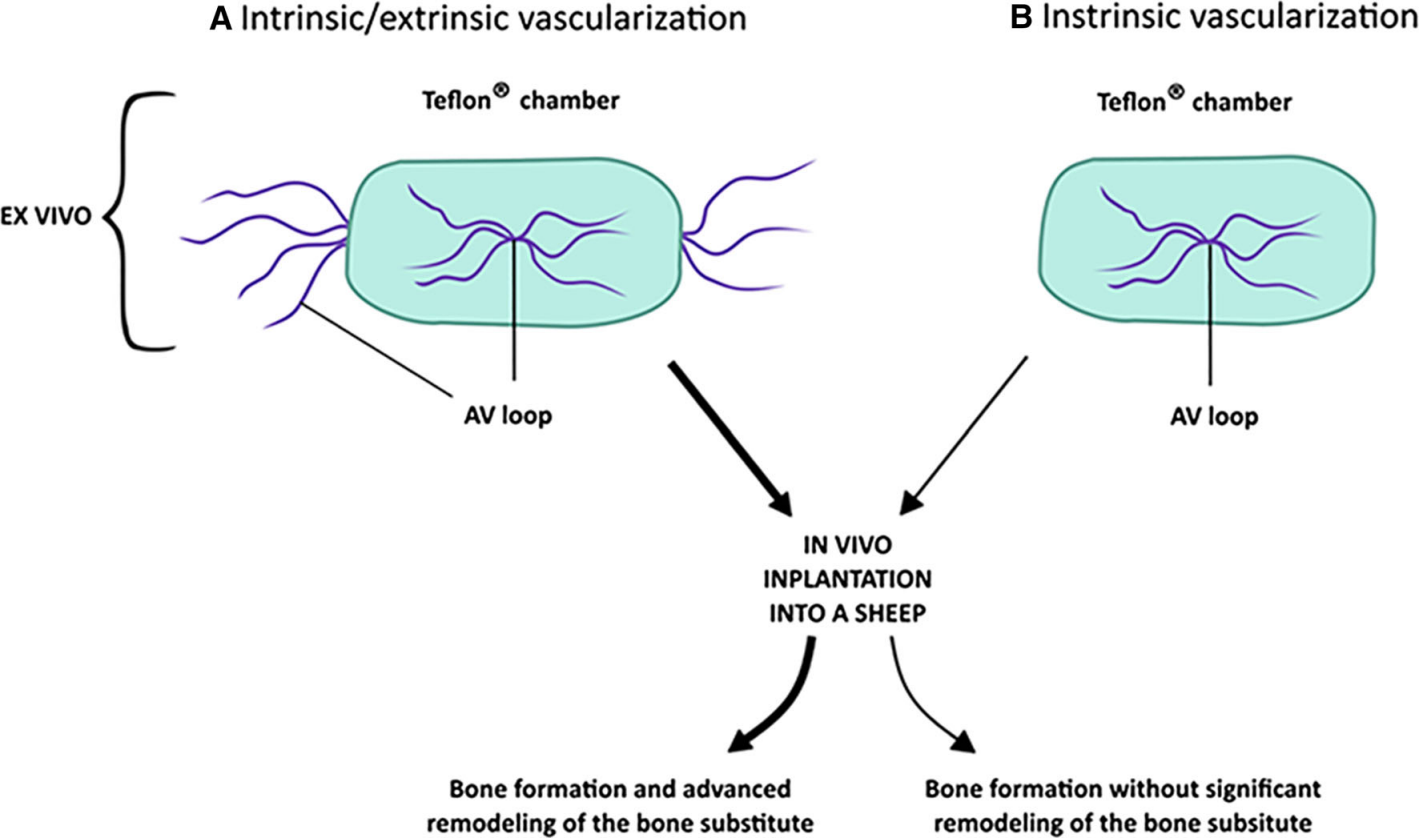
Effects of the intrinsic/extrinsic (**a**) and intrinsic (**b**) vascularization modes on in vivo bone formation and remodeling by the bone substitutes composed of Teflon^®^ chambers. Based on [82]

Not only is the existence of vascular network indispensable for a proper bone formation, but it is also crucial for its functional restoration in a disease or trauma-suffering patients. It is believed that the presence of blood vessels within the autologous bone grafts is critical for a successful bone regeneration, e.g., in the course of femoral head osteonecrosis therapy or post-oncological bone reconstruction [76, 77]. Although bone grafting serves as a gold standard in bone defect treatment, its application is limited [78] and thus forces elaboration of some new, alternative strategies, e.g., based on bone tissue engineering. On the other hand, appropriate bone-regenerating constructs strongly rely on a successful vascularization [79]. Proper blood supply is especially important in case of functional bone constructs, possessing a clinically relevant size, which leads to greater demands for oxygen and nutrients compared to small bioimplants [80]. Blood vessel supply within the tissue-engineered implants may positively influence the process of constructs’ osseointegration and bone defect restoration [81]. Recent studies point to a significant role of vascularization techniques in bone tissue engineering. For instance, the group of Weigand et al. [82] hypothesized that extrinsic and intrinsic vascularization of a large size bone tissue construct determines a successful regeneration process within a bone defect. This study was carried out on sheep. The authors created microsurgically an arteriovenous (AV) loop and connected it with the bone substitute in either perforated titanium chambers (intrinsic/extrinsic) for different time intervals of up to 18 weeks, or tested the isolated Teflon^(®)^ chambers (intrinsic) for 18 weeks (Fig. 4 **)**. A newly formed bone tissue was observed as early as 12 weeks post-implantation within the bioimplants vascularized by means of the intrinsic/extrinsic method. In the intrinsic vascularization model, degradation of the scaffold and osteoclastic activity was significantly lower after 18 weeks, compared to the combined intrinsic–extrinsic model, where degradation and osteoclast resorptive activity was observed as early as 12 weeks post-implantation. An increase in bone tissue formation after 18 weeks confirmed with the immunohistochemical staining for collagen type I, alkaline phosphatase and osteocalcin, was comparable for both the intrinsic/extrinsic and intrinsic vascularization models.

Modern strategies of bone defect regeneration can also be based on gene therapy, targeting different molecular signaling pathways involved in both angiogenesis and osteogenesis.

For example, Zhang et al. [83], demonstrated that adeno-associated viral vectors carrying the sequences encoding VEGF-A-the main factor involved in angiogenesis and BMP-7, which participates in osteogenesis can effectively enhance healing of the femoral head in a rabbit model of early, steroid-induced avascular necrosis of the femoral head (SANFH). A combination of those two sequences in one vector was more effective compared to a separate expression of either VEGF-A or BMP-7. Application of a gene therapy-based approach in the treatment of the femoral head necrosis has also been tested by Cao et al. [84]. These authors used deproteinized bone (DPB), absorbed with recombinant plasmid pcDNA3.1-hVEGF165, carrying the sequence coding for VEGF-A. This DPB-VEGF compound was further implanted into the drilled tunnel of a necrotic femoral head in rabbits.

Cao et al. also assessed the effects of DPB alone and compared its performance to the control group, i.e., a tunnel drilled in the necrotic femoral heads, left untreated. The authors concluded that a transfection of hVEGF165 gene promotes local angiogenesis and that a combination of DPB with VEGF improves the repair of the necrotic femoral head. Therefore, a combination of DBP and pcDNA3.1-hVEGF165 may become a potential, alternative method for the treatment of osteonecrosis.

## Summary

Blood vessels supplying bones orchestrate the process of bone development and remodeling as well as regulate the skeleton regeneration by delivering the nutrients, oxygen, hormones or growth factors to the bone cells. Blood vessels localized in the bone marrow cavity of the long bones also coordinate the process of hematopoiesis and provide the niches occupied by the precursors of blood cells.

Recent findings point to a regulatory role and molecular crosstalk between bone-related endothelium and osteoblasts, which play a significant role in bone development and regeneration. These processes are regulated mainly through the secretion of VEGF, the most important factor of angiogenesis. Mutual communication between the bone cells and endothelium is especially vital for a proper course of osteogenesis. In this context, skeletal blood vessels were shown to be lined with highly specialized endothelial cells.

The association between the proliferation and differentiation of bone precursors and the activity of type H endothelial cells, secreting Noggin has been particularly well documented. Also, a new role of bone blood vessel endothelium during bone mineralization has been recently revealed. This knowledge may have some relevant clinical implications for the treatment of bone regeneration disorders or bone loss, e.g., in older people, often suffering from osteoporosis or avascular osteonecrosis. However, it is currently believed that bone blood vessels may also be impaired by different systemic diseases: diabetes mellitus or atherosclerosis—which, by affecting a whole body, influence the health of the skeletal system. Both skeletal and systemic diseases may result from either a disturbance of a general blood vessel network or of the bone-specific vasculature itself. Aging also negatively affects bone vasculature, and these effects seem to be intensified in systemic diseases.

Not only is proper vascularization indispensable for bone formation or remodeling, it is a key factor in development of the in- and ex vivo bone regeneration strategies, e.g., based on tissue engineering. Thus, the knowledge and capability to target the molecular mechanisms underlying bone blood vessel functioning may deliver us with some new strategies to treat the vasculature-dependant skeletal and systemic diseases which negatively affect the bone metabolism.
